# Insights from the molecular docking analysis of colistin with the PmrA protein model from Acinetobacter baumannii

**DOI:** 10.6026/97320630018041

**Published:** 2022-01-31

**Authors:** Shalini Ganeshan, Mohammad Reza Shakibaie, Rajaguru Rajagopal

**Affiliations:** 1 Department of Biotechnology, Tips college of arts and science, Coimbatore, Tamil Nadu 641107, India; 2 Department of Microbiology and Virology, Kerman University of Medical Sciences, Kerman, Iran; 3 Environmental Health Engineering Research Center, Kerman University of Medical Science, Kerman, Iran; 4 Research Scholar Department of Pharmaceutics, Mother Theresa Post Graduate and Research Institute of Health Sciences, Pondicherry-605006, India

**Keywords:** Acinetobacter baumannii, protein modeling, model quality, molecular docking

## Abstract

Acinetobacter baumannii (AB) is one of the most common causes of nosocomial infections. Therefore, it is of interest to design and develop drugs against Acinetobacter baumannii. A strain of AB showing MIC 32 µg/ml against colistin was isolated from a hospital environment in Iran. Hence, we document data to glean insights from the molecular docking analysis of colistin with the PmrA protein from this bacterium.

## Background:

Acinetobacter baumannii is one of the most common causes of nosocomial infections in intensive care units (ICUs) of different hospitals across the globe and its ability to acquire diverse mechanisms of antibiotic resistance limits the therapeutic choices for the treatment of immuno compromised and critically ill patients in ICUs [1,2]. Nevertheless, resistance to other antimicrobial drugs such as tigecycline, ampicillin-sulbactam, and ertapenem are emerging rapidly [3]. This is true even with colistin, whichis the last-resort antibiotic against carbapenemase-producing strains ofAB [4]. However, the outbreaks of colistin-resistant infections are common [4-6]. The increasing importance of the species has been recognized by the WHO which classified carbapenem-resistant A. baumannii (CRAB) [7]. The colistin displaces competitively divalent cations (calcium and magnesium) from the negatively charged phosphate groups of membrane lipids usually cross-bridge adjacent LPS molecules necessary for membrane stabilization [8]. Mutations in either pmrA or pmrB genes have been associated with colistin-resistance in Gram-negative organisms such as Klebsiella pneumoniae, A. baumannii and Pseudomonas aeruginosa [9]. Data shows that the PmrA protein from A. baumannii is a potential drug target [10-15]. The most common mechanism of acquired resistance to colistin in the AB involves modification of the LPS component of the outer membrane, specifically the 1' and 4' phosphate groups of lipid A which neutralize the net negative charge group and reduce binding to colistin [16]. Inhibition of the PmrA with little molecules or colistin may potentially block PmrC over expression and cut the colistin resistance pathway [17]. Therefore, it is of interest to document the molecular docking analysis data of colistin with the PmrA protein.

## Materials and methods:

### Bacterial sources, antimicrobial susceptibility and detection of pmrA gene:

More than 68 A. baumannii strains were isolated from different wards of two main hospitals in Kerman, Iran during a one-year period.Biochemical and genetic characteristics of PmrA and PmrB were studied as described elsewhere [4].

### Sequence accession number:

The PmrA protein sequence was deposited in the GenBank (NCBI) and UniProtKB/TrEMBL databases under accession numbers QIC34671 and A0A6C0W633, respectively [4].

### Assessment of the physicochemical parameters of PmrA:

Physicochemical parameters of the PmrA protein such as molecular weight, theoretical isoelectric point, atomic composition, extinction coefficient, instability index, and aliphatic index, the amino acid composition and grand average of hydropathicity were determined using Expasy's ProtParam server (http://web.expasy.org/protparam/).

### Classificationand functional analysis of PmrA:

The primary classification and functional analysis of PmrA were completed usingInterPro software version 5.51-85 (www.Ebi.ac.uk/interpro/) which provides an integrative classification of protein sequences into the family, superfamily, and domains. The biological functions of the PmrA protein, including ligand-binding site, molecular and cellular functions in terms of Gene Ontology (GO) were inferred from the PDP database [18].

### Structural prediction of PmrA and stability:

The 3D structure of PmrA was created from the primary amino acid sequence using the I-TASSER platform with Monte Carlo simulations (https://zhanggroup.org/I-TASSER/). The functional domains of PmrA were determined using the ROSETTA prediction tool (https://www.rosettacommons.org/) and Hidden Markov Model program (https://www.ebi.ac.uk/Tools/hmmer/) with atomic-level accuracy of more than 2.5 Å. The stability of the models was validated using the ProSA algorithm. The predicted models were then subjected to the orientation of dihedral angles including phi (ϕ) and psi (ψ) and backbone conformation using the PROCHECK module of the PDB Sum server (https://www.ebi.ac.uk/thornton-srv/software/PROCHECK/) and Ramachandran plot (https://saves.mbi.ucla.edu/). The quality of the generated models was estimated using the Z-score of LOMETS threading alignments as mathematically formulated elsewhere [19].

### Domains analysis and accuracy:

ROSETTA (https://www.rosettacommons.org/), InterPro software version 5.51-85 (www.Ebi.ac.uk/interpro/) and Pfam database were used for domain analysis.

### Detection of the domain boundaries:

FUpred (Folding Unit predictor) was used to detect domain boundaries from protein sequences based on contact map prediction. The secondary structure of a sequence is predicted using PSSpred, and the contact map (with Cβ-Cβ distance <8 Å) wasestimated using the ResPRE method. The contact map and secondary structure data are used to calculate the FUscore [20].

### Receptor grid generation:

Receptor grid boxes were generated using "Glide (Grid-based Ligand Docking with Energetics) in a protein preparation wizard of the Maestro program [21]. Refinement of the structure was completedusing the OPLS-2005 force field energy minimization program [22]. The calculation was performed by the chemical transformation of reference ligand into target ligandusing GROMACS package [23]. The grid was generated using the Receptor Glide 4.0 XP module based on an area of interaction between the target protein and ligand molecule in terms of X, Y, and Z coordinates.

### Ligand preparation:

Colistin ligand was retrieved from the PubChem database and prepared using the LigPrep module in Maestro v11 [24]. The highest binding and corresponding interactions pose were visualized and inspected in PyMOL (https://www.schrodinger.com/products/pymol). The energy minimization was completed at neutral pH 7.0 ± 2.0 using the least square OPLS_2005 force field [22].

### Molecular docking analysis and virtual screening:

The favorable interactions between the selected ligands molecules and modeled receptor were identified using the extra precision (XP) feature of Grid-based Ligand Docking with Energetics (GLIDE, a Schrödinger module) [25]. Ligands were energy minimized using the Universal Force Field (UFF) and converted to pdbqt format in PyRx0.8 for virtual screening [26]. Virtual screening was performed using PyRx0.8 with VINA and the predicted binding affinity was calculated in kcal/mol [27]. Binding sites were generated using the SiteMap tool. The best docking poses were selected directly using the Glide G-Score as described elsewhere [28]. Optimal binding free energy and the various sequence features that distinguish sequence from the reference sequence (usually taken to be the optimal sequence) was also calculated [28].

## Results and Discussion:

**Figure 1:**
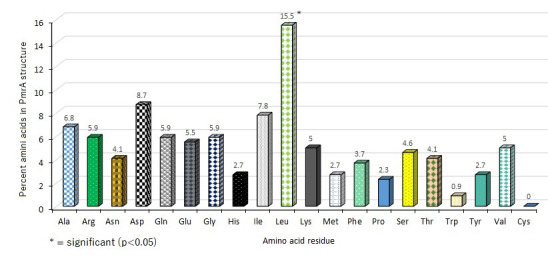
Amino acids composition of A. baumannii colistin resistant PmrA protein calculated using the Expasy's ProtParam server. Leu with 15.5% was the most abundant amino acid detected and Cys was absent.

**Figure 2:**
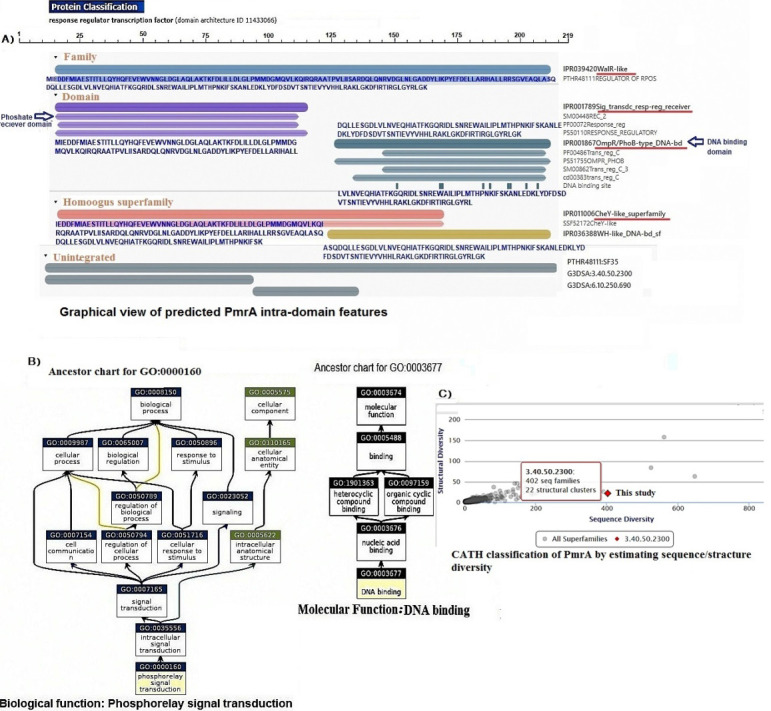
A) The classification of PmrA based on domains, family and homologues superfamily using InterPro protein viewer, B) The biological and molecular functions of PmrA protein using Gene Ontology (GO), C) The sequence/structure diversity of OmpR/PhoB superfamily of PmrA compared to the other homologues superfamilies in CATH (Class Architecture Topology Homologous superfamily) database.

The genetically characterized PmrA sequence from a colistin-resistant A. baumanniiwas downloaded from the GenBank database with accession numbers QIC34671 as described elsewhere [29]. The physicochemical characteristics, Gene Ontology, molecular docking, and domain boundaries of PmrA protein were analyzed. The results of the Expasy InterPro server revealed that the PmrA was a monomeric protein containing 219 amino acids which contributed to an average molecular weight of 24961.72 kDa. The physicochemical parameters of the PmrA are listed in Table 1(see PDF). The protein was classified as stable with an instability index value of 27.98, acidic (pH-5.6) with an aliphatic index of 112.24, and extinction coefficient 0.97. It had a grand average hydrophobicity (GRAVY) of -0.139. Gill et al. [30] presented a method for calculating the accurate (to +/- 5% in most cases) molar extinction coefficients for proteins from amino acid composition. Aliphatic amino acid side chains of PmrA were responsible for the increase in thermal stability. The aliphatic index values of an antifreeze protein ranges from 57.83 to 125.23 [31] and it is similar to PmrA. Therefore, PmrA protein is stable in a wide temperature range. Hierarchical clustering analysis of the PmrA molecule revealed it was belonged to the transcription regulatory Protein WalR-Like superfamily, CheY family and member of OmpR/PhoB transcriptional response regulator. Furthermore, the composition of PmrA protein differed from reported the data in the PDP database with more Leu (15.5%), ASP (8.7%), and Ile (7.8%) as shown in Figure 1. The analysis of domains by InterPro, ScanProsite and Pfam revealed that the PmrA A0A6C0W633 consisted of two main domains, one was in the N-terminal regulatory domain (REC) with sequence span 1 - 111 and score = 39.208. The C-terminal contained OmpR/PhoB-type DNA-binding domain (DBD) with sequence span 124 - 218 and score = 34.601 and ASP with starting codon at position 124 (Figure 2A).

Hierarchical data based on Gene Ontology including biological functions and molecular activities illustrated in Figure 2B. There was significant diversity in the biological activity of PmrA protein such as a signal transduction system, cell communication, regulation of transcription, and cellular response to a foreign stimulus. The molecular function is linked to DNA binding ability and transcriptional control attributed indirectly to colistin-resistance (Figure 2B). Nevertheless, the sequence / structure diversity of this superfamily compared to other superfamilies in CATH (Class Architecture Topology Homologous Superfamily) database showed 24 structural clusters (Figure 2C). It also showed 75% sequence/structure homology with the other two components regulatory family.

**Figure 3:**
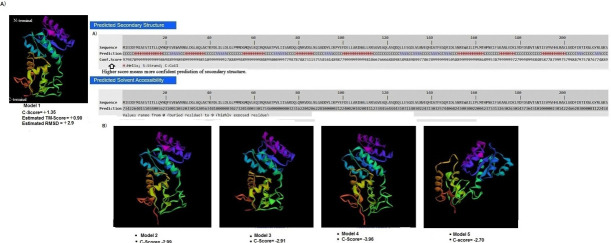
A) Predicted secondary structure including number of helices, strand and coil and solvent accessibility of Pmr A, B) Top 5 final models of PmrA protein generated using I-TASSER. The predicted model quality was assessed using the calculation of C, TM and RMSD parameters from LOMET. N-terminal region of PmrA is shown in blue color and C-terminal depicted in red color.

**Figure 4:**
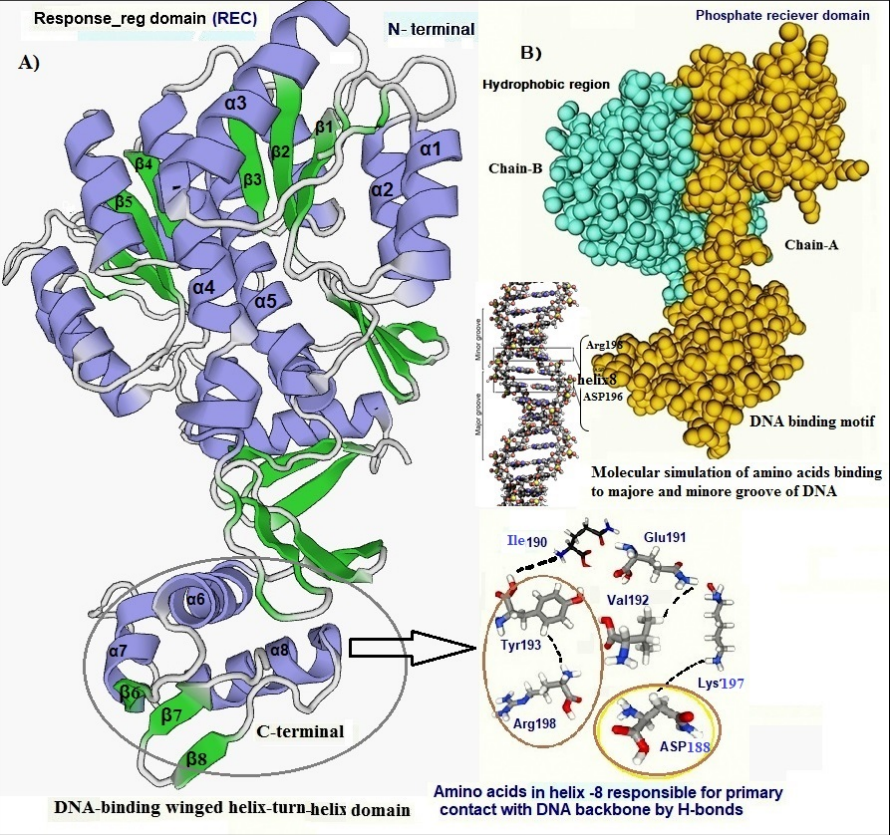
A) Cartoon structural representation and number of chains in the PmrA protein and the key amino acids involved in DNA binding domain, B) Molecular simulation and spacefil structure model of PmrA and its interaction with the DNA molecule.

**Figure 5:**
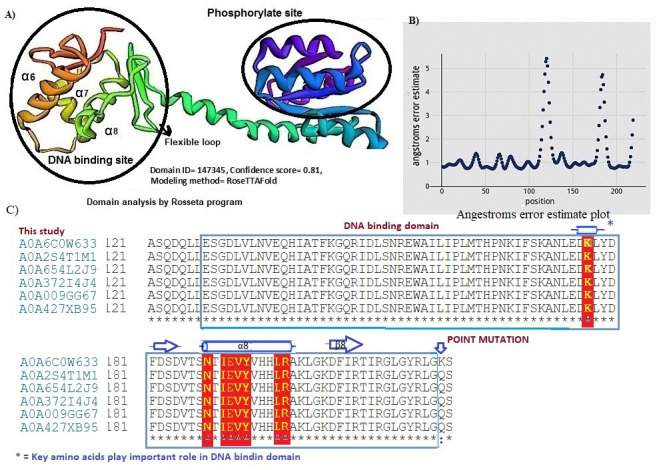
A) Stereochemistry of alpha 8 involved in DNA binding activity by ROSETTA software, B) Angstrom error plot estimate of PmrA molecule, C) Multiple amino acids alignments of BDB domain of PmrA with closely sequences in UniProtKB database. The conserved amino acids which are important in domain binding are shown in yellow color.

**Figure 6:**
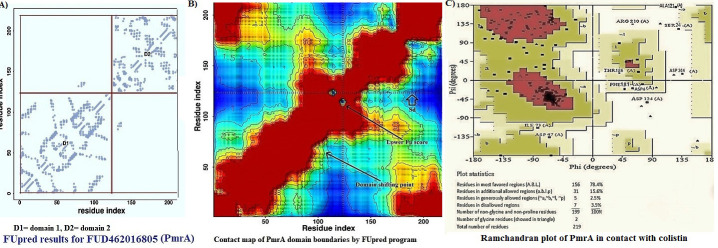
A) The distogram of contact map of PmrA using LOMESTS program. Color scale represents a distance of 1-20+ angstroms, B) Contact map boundaries of PmrA protein using FUpred program, C) The logistic representation of PmrA using Ramachandran plot by rampage.

**Figure 7:**
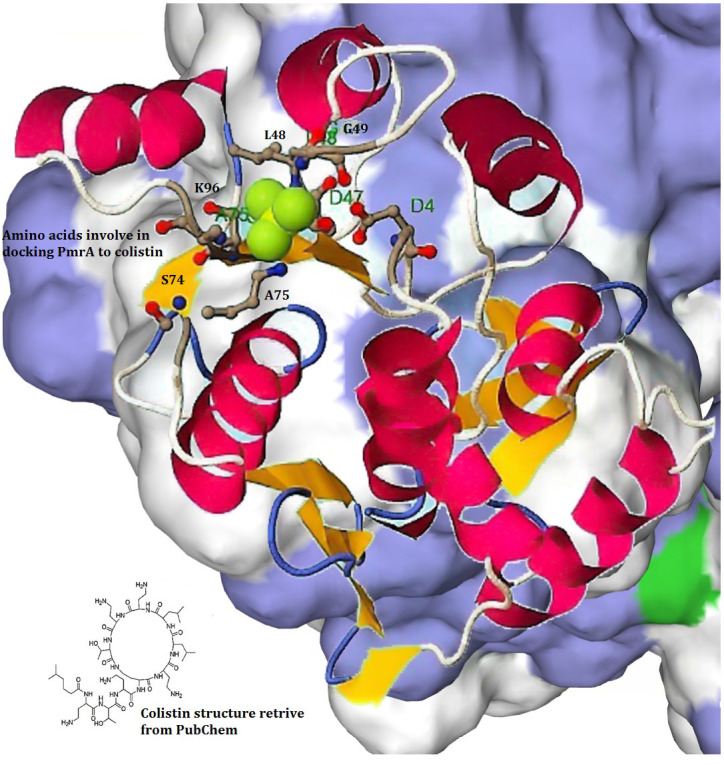
Molecular docking of pmrA with colistin and key amino acids involved in this process. The colistin was more effective with the active site residues ASP 4, ALA 75, SER 74, and LYS 96, respectively.

The secondary structure and solvent accessibility of PmrA by the I-TASSER platform are shown in Figure 3A. The confidence prediction score (C-score) of PmrA conformation was around 8-9. Solvent accessibility values from 0 (buried residue) to 8 (highly exposed residue) was also calculated. Five closely related models were selected with each having a C-Score of +1.35, -2.99, and -3.96, respectively (Figure 3B). Model 1 with C-score +1.35, RMSD +2.9, and TM 0.90 ±0.06 was selected as the target model for further analysis. Nevertheless, the PmrA Z score was -6.02 (Figure 3B). We also analysed the protein conformation in the form of an α/β sandwich both in the N-terminal and C -terminal regions (Figure 3A). Luo et al. [32] cloned PmrA from K. pneumoniae into a pET-29b vector and subjected to crystallization. The N-terminal containing a head-to-head REC dimer displaying the alpha 4–beta 5–alpha 5 interface is shown. The predicted 3D protein structures can be as close as 1-2Å root mean square deviation (RMSD) to their native structures for proteins with close homologous templates [34]. The predicted PmrA secondary structure was found to be containing 13 α-helix and 16 β-strands connected by coils. The PmrA contains two domain (phosphate receiver domain (REC) and the C-terminal DNA binding domain DBD). Each activated REC contains a five-stranded parallel β-sheet (β 1-5) surrounded by five α-helices (α1-5) (Figure 4A). The DBD contains 3 β-sheet (β 6-8) and three α-helix (α 6-8). Several residues spanning the C-terminal form H-bonds with a DNA phosphate backbone through Arg198 and Asp 188 on the helix α8 is shown in Figure 4A. We found Arg198 and Asp188 of a8 play an important role (Figure 4B). It has been reported in A. baumannii the DBD contains a β-sheet (β6-8), a central three-helix core (α6-8) and a C-terminal β-hairpin (β9-10) [33]. The DNA binding domain conformation displayed a head-to-tail orientation packed by three α helices against three β sheets. The key amino acids involved in the DNA binding domain showed a winged-helix motif (Figure 4A). They connected through H-bonds to several other amino acid residues of the helices a8 such as Ile 190 and Glu 191. These charged residues were stabilized by the formation of salt bridges among Arg198, Lys 197, and His 195 in the Free State. The conformation analysis using the ROSETTA program (Figure 5A) displayed angstrom error indicating minimum error in torsion around each amino acid residue resided except at positions 130-133 and 178-180, respectively (Figure 5B). Primary sequence alignment of C-terminal DNA binding domains of PmrA and five other response regulator proteins are showed in Figure 5C. The key amino acids in this domain showed in yellow color were well conserved. We used FUscore analysis by shifting the C-terminal contact map to the N-terminal for analyzing the discontinuous two-domain protein boundaries. A discontinuous two-domain of PmrA protein is shown in Figure 6A using FUscore described by Zhang et al. [34]. It was found that PmrA domain overlapping scores of FUpred were 0.839 and 0.672 which were higher than the control. The contact maps derived from the application of a distance cutoff of 9 to 11Å around the Cβ atoms constitute the most accurate representation of the visualized 3D structure (Figure 6A). The contact map of PmrA domain boundaries revealed the point of domain shifting score and lower FUpred region with Sd cross line as shown in Figure 6B. The quality of the predicted model structure was assessed using the Ramachandran plot generated by Rampage (Figure 6C). The plot showed only 3.5% in disallowed regions. The docking of the PmrA protein with the colistin molecule is shown in Figure 7. The colistin antibiotic was retrieved from the PubChem database (PubChem CID-5311054) using Glide (Glide is a ligand docking program for predicting protein-ligand binding modes and ranking ligands via high-throughput virtual screening). Graphical representations of the PmrA indicated that most of the interactions of colistin were with the amino acid residues ASP 4, GLY 49, LYS 96, and ALA 75 of the PmrA (Figure 7). Based on the hydrophobic interactions, hydrophobically packed H-bonds, lipophilic, low molecular weight, and electrostatic interactions, the glide scores were generated. The compound showed a G-Score of -2.41 Kcal/mol and seven H- bonds.This interactive site was located in the core region of the phosphate receiver domain.

## Conclusion:

We document the insights gleaned from the molecular docking analysis data of colistin with the PmrA protein model obtained from colistin-resistant A. baumannii for further consideration in drug discovery and development.
